# The resurgence of malaria in Northern Iran in 2023: a wake-up call

**DOI:** 10.1186/s12879-025-11266-x

**Published:** 2025-07-15

**Authors:** Faramarz Koohsar, Roghiyeh Faridnia, Ogholniaz Jorjani, Mohammad Taher Hojjati, Ganesh Yadagiri, Ghasem Noshak, Hosein Tavakoli Pirzaman, Hamed Kalani

**Affiliations:** 1 Laboratory Sciences Research Center, Golestan University of Medical Sciences, Gorgan, Iran; 2 Department of Veterinary Biosciences, The Ohio State University, Columbus, OH USA; 3 Infectious Diseases Research Center, Golestan University of Medical Sciences, Gorgan, Iran

**Keywords:** Golestan province, Malaria, North Iran, *Plasmodium falciparum*, *Plasmodium Vivax*

## Abstract

**Background:**

Control and elimination programs for malaria in Iran have consistently faced significant challenges due to various factors, including the presence of neighboring malaria-endemic countries such as Afghanistan and Pakistan. In recent years, Golestan Province in northern Iran has recorded few cases of imported malaria, with a sudden increase of 22 cases in 2023. This article provides an overview of the malaria situation in northern Iran, along with a detailed report of these 22 cases.

**Methods:**

The study population comprises all individuals exhibiting symptoms suspected of malaria (*n* = 445) who were referred to the Malaria Diagnosis Center in Golestan Province, located in northeastern Iran, for diagnosis between March 23, 2023, and December 23, 2023. A drop of peripheral blood, collected from a finger prick of each patient, was utilized for *Plasmodium falciparum*/*P*. *vivax* antigen detection. Moreover, thin and thick smears were prepared for each patient to investigate morphological characteristics and parasitemia percentage of the parasites.

**Results:**

In total, 4.94% (22/445) of individuals with malaria-suspected symptoms were infected with *Plasmodium* species. All 22 infected individuals were native to Golestan Province, and none had a previous history. The mean ± standard error of mean (SEM) for the number of parasites/µL of blood sample was 16,029 ± 5,060 for *P*. *vivax* and 105,460 ± 102,146 for *P*. *falciparum*. Among the patients, 77.27% (17/22) were infected with *P*. *vivax*, 18.18% (4/22) were infected with *P*. *falciparum*, and 4.54% (1/22) were co-infected with both *P*. *vivax* and *P*. *falciparum*. In the case of *P*. *falciparum*, 40% (1/5) of the samples presented the ring form, 60% (3/5) trophozoite form, and 20% (1/5) gametocyte form. All patients demonstrated a positive response to the treatment, with a decrease in both the number of parasites and the number of clinical symptoms over time.

**Conclusions:**

This study discussed 22 cases of malaria diagnosed in 2023 in Golestan Province in northern Iran. Given the presence of malaria vectors in this area and the observation of gametocytes in specimens from some patients, the increase in reported malaria cases could be worrisome in terms of establishing local transmission in this area.

## Background

Malaria is a disease caused by a protozoan parasite belonging to the genus *Plasmodium*, which is transmitted through the bite of a female Anopheles mosquito [1]. The World Health Organization estimated that there were 249 million cases of malaria globally in 2022, representing an increase of 5 million cases from the previous year. The majority of reported cases and deaths due to malaria are concentrated in Africa, accounting for 93.6% of cases and 95.4% of deaths worldwide [2].

While Africa remains the region most affected, there has also been a concerning rise in malaria cases in other parts of the world, such as the Middle East. In countries of South and Southwest Asia, including Pakistan and Afghanistan—bordering Iran—a significant 34% increase in malaria cases was observed from 2021 to 2022. In 2022, Iran reported 1,439 indigenous malaria cases, indicating a resurgence of the disease in the region. However, due to the absence of data on indigenous or introduced malaria cases between 2018 and 2021, the current transmission dynamics and endemic status of malaria in Iran remain unclear [3].

The northern region of Iran, situated along the Caspian Sea coast and encompassing the provinces of Golestan, Mazandaran, and Gilan, has historically been a hyperendemic area for malaria, with documented outbreaks in the past. Notably, the Caspian Sea coast has experienced the largest malaria outbreak in Iran, resulting in over 20,000 fatalities [4]. Understanding recent trends in malaria incidence in Golestan Province is crucial for guiding local public health interventions and maintaining progress toward malaria elimination.

This study aims to provide a comprehensive overview of the evolving landscape of malaria in northern Iran, with a specific focus on new cases reported in Golestan Province.

## Materials and methods

### Study population

The study population comprises all individuals exhibiting symptoms suspected of malaria (*n* = 445; 182 females and 263 males) who were referred to the Malaria Diagnosis Center in Golestan Province, located in northeastern Iran, for diagnosis (passive detection) between March 23, 2023, and December 23, 2023.

### *Plasmodium* antigen detection

A drop of peripheral blood, collected from a finger prick of each patient, was utilized for diagnosis. This test was conducted via the First Response^®^ Malaria Ag *Pf*/*Pv* Card Test following the manufacturer’s instructions. The kit utilizes two monoclonal antibodies: one targeting lactate dehydrogenase specific to *P*. *vivax* and the other targeting histidine-rich protein 2 (HRP2) of *P*. *falciparum*. This kit is approved by the World Health Organization and is classified as a rapid diagnostic test (RDT) [5].

### Giemsa staining

To prepare thin smears, a small drop of blood was placed onto one end of a clean glass slide and spread evenly across the slide to create a thin film. The smear was allowed to air dry completely and fixed by applying a small amount of methanol for approximately 1 to 2 min, after which it was stained with Giemsa stain. To prepare thick smears, 2 to 4 drops of blood were placed onto a clean glass slide and spread onto a thick film approximately 1 to 2 mm in diameter. The smear was allowed to air dry completely without fixation and then stained with Giemsa stain [6, 7]. The *Plasmodium* species and parasite density were determined [8, 9]. For each patient, two experts under light microscope independently examined two smears.

### Data analysis

The data were analyzed via IBM SPSS v20 software and Fisher’s exact statistical test. The results were considered significant when the *P* value was less than 0.05.

## Results

**Fig. 1:**
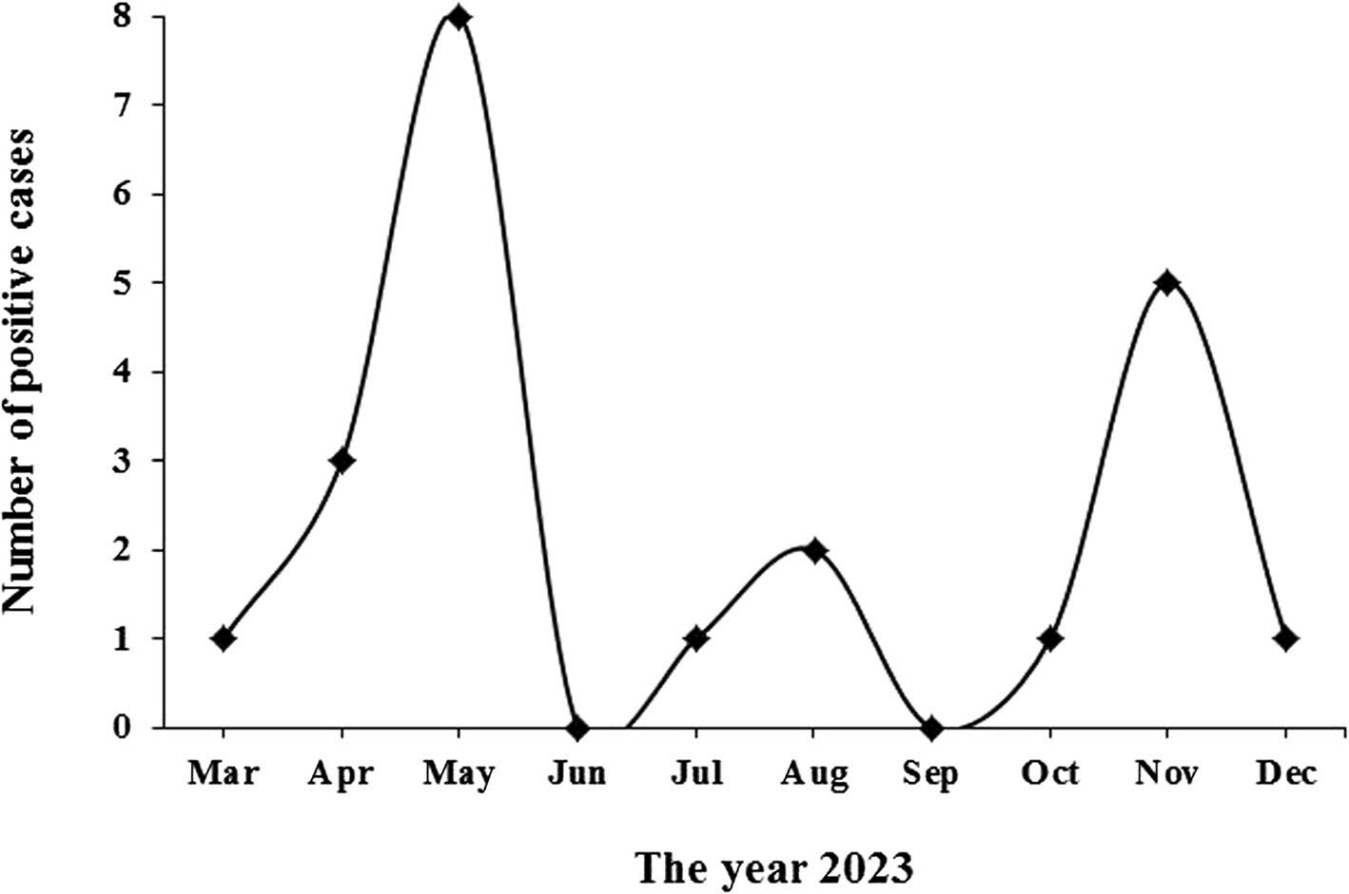
Malaria trends in different months of 2023 in Golestan Province, northern Iran (*P* = 0.02; using Fisher’s exact statistical test)

In total, 4.94% (22/445) of individuals with malaria-suspected symptoms were infected with *Plasmodium* species. These cases were detected as passive in Golestan Province, northern Iran (Table 1). The trend of the disease across different months of the year is illustrated in Fig. 1 (*P* = 0.02). The clinical symptoms observed in patients included fever (100%; 22/22), sweating (95.45%; 21/22), chills (86.36%; 19/22), myalgia (86.36%; 19/22), headache (81.81%; 18/22), and nausea (40.9%; 13/22).

**Table 1:** Information approximately 22 cases of imported malaria in Golestan province, Northern iran, in 2023

Number	Occupation	City of Residence	Nationality	Past infection	Travel^a^	Symptoms^b^	Onset of symptoms	Case detection	Medical care^c^	Species	Parasite/µL of blood sample	Severity of parasitemia^d^	Parasite stage	Type of transmission	Treatment^e^
1	Driver	Aq Qala	Iranian	No	Iranshahr	F, S, Ch, M, H	March 23, 2023	Passive	I	*P*. *vivax*	14,000	Intermediate	Trophozoite, Schizont	Imported	CQ-PQ
2	Military	Bandar-e Torkeman	Iranian	No	Saravan	F, Ch, N	April 15, 2023	Passive	I	*P*. *vivax*, *P*. *falciparum*	10,600	Intermediate	Trophozoite	Imported	AS-SP
3	Military	Gorgan	Iranian	No	Saravan	F, S, Ch, M	April 21, 2023	Passive	O	*P*. *vivax*	11,000	Intermediate	Trophozoite, Schizont	Imported	CQ-PQ
4	Military	Ramian	Iranian	No	Saravan	F, S, Ch, M, H, N	April 29, 2023	Passive	O	*P*. *vivax*	5,500	Low	Trophozoite, Gametocyte	Imported	CQ-PQ
5	Military	Gonbad-e Kavus	Iranian	No	Saravan	F, S, Ch, M, H, N	May 9, 2023	Passive	I	*P*. *vivax*	9,260	Low	Trophozoite	Imported	CQ-PQ
6	Military	Gorgan	Iranian	No	Saravan	F, S, Ch, H	May 17, 2023	Passive	O	*P*. *vivax*	10,500	Low	Trophozoite	Imported	CQ-PQ
7	Military	Gorgan	Iranian	No	Saravan	F, S, Ch, M, H, N	May 17, 2023	Passive	O	*P*. *falciparum*	600	Low	Ring form	Imported	AS-SP
8	Military	Gorgan	Iranian	No	Saravan	F, S, Ch, H	May 17, 2023	Passive	O	*P*. *vivax*	338	Low	Trophozoite	Imported	CQ-PQ
9	Military	Gorgan	Iranian	No	Saravan	F, S, M, H	May 18, 2023	Passive	O	*P*. *vivax*	17,090	Intermediate	Trophozoite	Imported	CQ-PQ
10	Self-employed	Bandar-e Gaz	Iranian	No	Saravan	F, S, Ch, M, H, N	May 19, 2023	Passive	O	*P*. *falciparum*	8,436	Low	Trophozoite, Gametocyte	Imported	AS-SP
11	Military	Gorgan	Iranian	No	Chabahar	F, S, Ch, M, H, N	December 23, 2023	Passive	O	*P*. *vivax*	20	Low	Trophozoite	Imported	CQ-PQ
12	Military	Gorgan	Iranian	No	Saravan	F, S, M, H	May 21, 2023	Passive	O	*P*. *vivax*	1,000	Low	Ring form	Imported	CQ-PQ
13	Military	Aq Qala	Iranian	No	Saravan	F, S, Ch, M, H, N	May 22, 2023	Passive	I	*P*. *vivax*	15,807	Intermediate	Trophozoite, Schizont	Imported	CQ-PQ
14	Driver	Gonbad-e Kavus	Iranian	No	Pakistan	F, S, Ch, M, H, N	July 2, 2023	Passive	I	*P*. *falciparum*	411,852	High	Trophozoite	Imported	AS-SP
15	Driver	Gorgan	Iranian	No	Pakistan	F, S, Ch, M, H, N	August 23, 2023	Passive	I	*P*. *vivax*	12,450	Intermediate	Trophozoite	Imported	CQ-PQ
16	Driver	Aq Qala	Iranian	No	Chabahar	F, S, Ch, M, H	August 25, 2023	Passive	O	*P*. *vivax*	79,600	Intermediate	Trophozoite	Imported	CQ-PQ
17	Driver	Aq Qala	Iranian	No	Chabahar	F, S, Ch, M, H, N	October 31, 2023	Passive	I	*P*. *vivax*	10,302	Intermediate	Trophozoite, Schizont	Imported	CQ-PQ
18	Driver	Aq Qala	Iranian	No	Chabahar	F, S, Ch	November 4, 2023	Passive	I	*P*. *vivax*	46,400	Intermediate	Trophozoite, Gametocyte	Imported	CQ-PQ
19	Military	Gorgan	Iranian	No	Saravan	F, S, Ch, M, H, N	November 5, 2023	Passive	O	*P*. *falciparum*	955	Low	Ring form	Imported	AS-SP
20	Driver	Bandar-e Torkeman	Iranian	No	Bandar-e Lengeh	F, S, Ch, M	November 11, 2023	Passive	I	*P*. *vivax*	238	Low	Trophozoite, Schizont, Gametocyte	Imported	CQ-PQ
21	Driver	Gonbad-e Kavus	Iranian	No	Pakistan	F, S, Ch, M, H, N	November 13, 2023	Passive	I	*P*. *vivax*	38,700	Intermediate	Trophozoite	Imported	CQ-PQ
22	Driver	Bandar-e Torkeman	Iranian	No	Bandar-e Lengeh	F, S, Ch, M, H, N	November 28, 2023	Passive	I	*P*. *vivax*	304	Low	Gametocyte	Imported	CQ-PQ

^a^Travel history over the last two months

^b^*F* fever, *S* sweating, *Ch* chills, *M* myalgia, *H* headache, *N* nausea

^c^*O* outpatient; *I* inpatient

^d^Low parasitemia: 5–10,000 parasites/µL; intermediate parasitemia: 10,000–100,000 parasites/µL; high parasitemia: > 100,000 parasites/µL [9]

^e^For *P*. *vivax* treatment: Chloroquine (CQ) 25 mg/kg for 3 days, including 10 mg/kg on the first day and 7.5 mg/kg on the second and third days; Primaquine (PQ) 3.5 mg/kg for 14 days, including 0.25 mg/kg daily. For *P*. *falciparum*: Artesunate (AS) for 3 days, 200 mg daily in two doses of 100 mg each along with 3 sulfadoxine-pyrimethamine tablets (500 mg sulfadoxine and 25 mg pyrimethamine) as a single dose with the first dose of artesunate

**Fig. 2:**
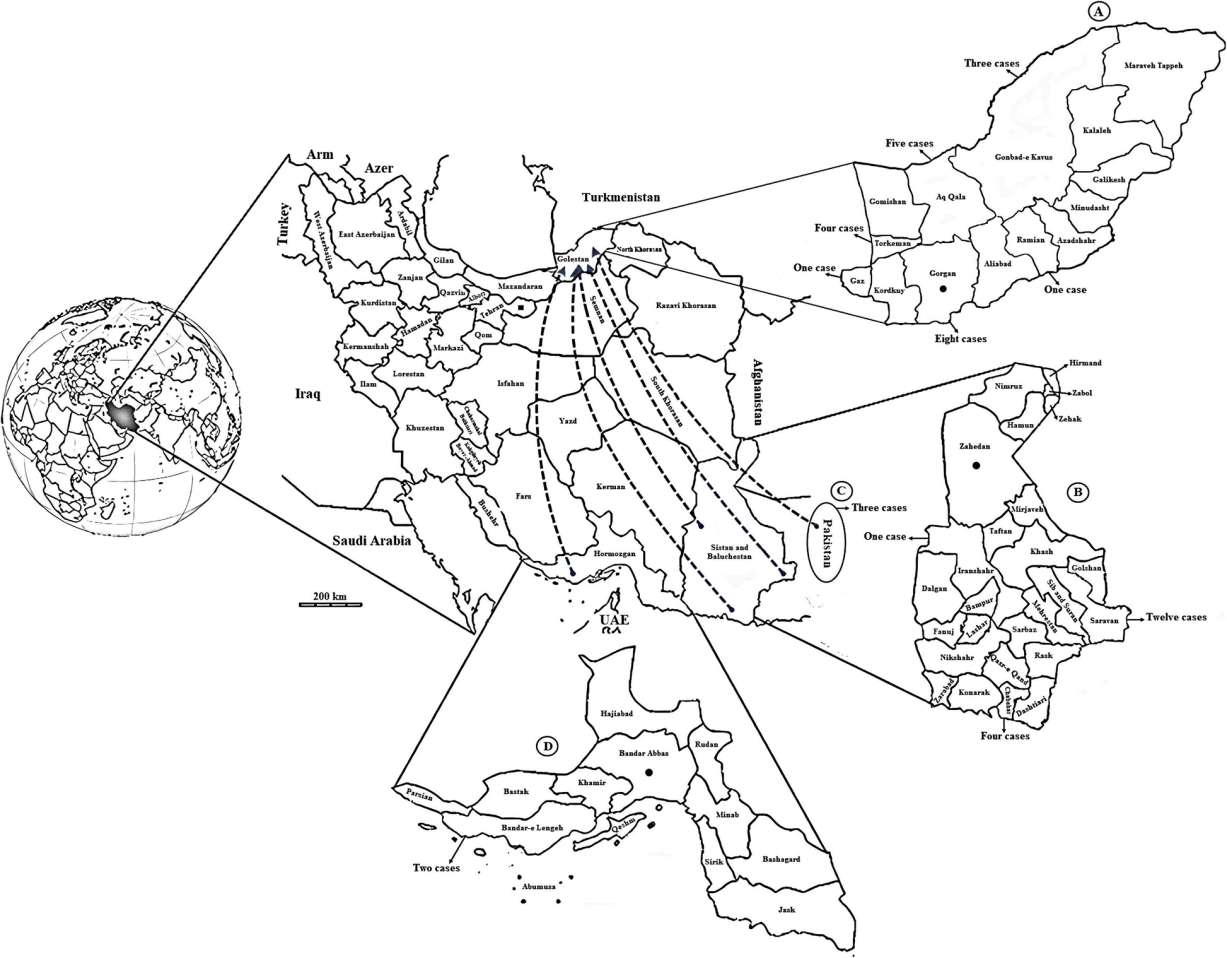
**A** Golestan Province, where 22 cases of imported malaria were identified. The gray areas show the residences of infected people and the number of cases identified from these areas. **B** Sistan and Baluchistan Provinces, where 17 infected people have traveled. The gray areas represent the cities to which the infected people traveled. **C** Pakistan, where 3 cases of infected people had traveled there. **D** Hormozgan Province, where two infected people have traveled. The gray area shows the city to which the infected people traveled. See Mazandaran and Gilan Provinces, which are located east of Golestan Province in northern Iran, and in this article, an overview of the malaria situation in these three provinces is presented

All the patients were male. The mean ± confidence interval of age in the infected individuals was 37.92 ± 8.74. Moreover, 54.54% (12/22) of the patients were military personnel, 40.9% (9/22) were drivers, and 4.54% (1/22) were self-employed (*P* = 0.007). All 22 infected individuals were native to Golestan Province, and none had a previous history (Fig. 2). The infected individuals reported a history of travel to Sistan and Baluchistan Provinces in southeastern Iran (77.27%; 17/22), Hormozgan Province in southern Iran (9.09%; 2/22), and Pakistan (13.63%; 3/22) (Fig. 2).

**Fig. 3:**
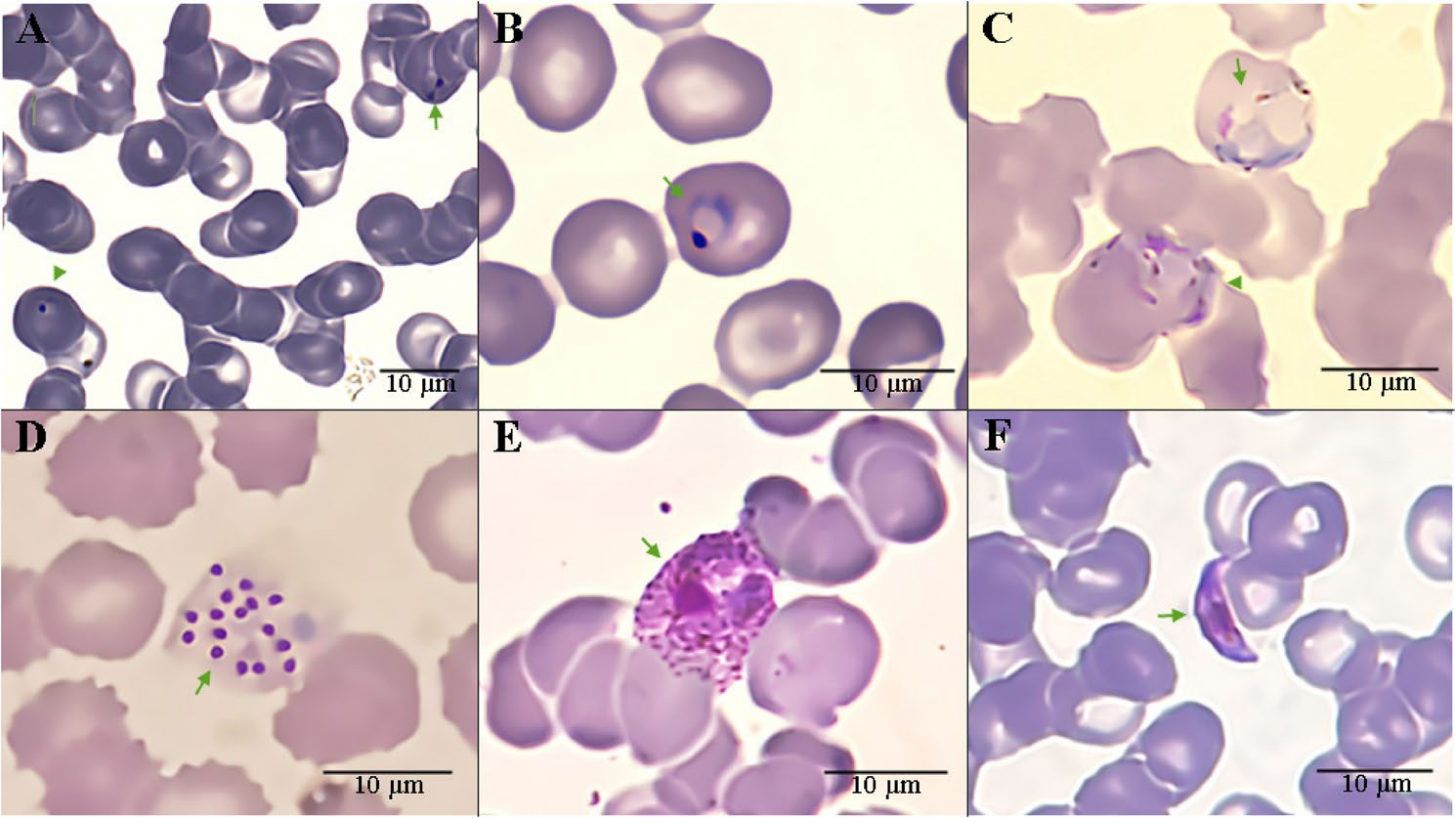
**A** Co-infection with *P*. *falciparum* and *P*. *vivax*. *P*. *falciparum* headphone-like trophozoite (arrow) and *P*. *vivax* ring form (arrowhead); **B** Growing trophozoite of *P*. *vivax* (arrow); **C** Amoeboid form (arrow) and early schizont of *P*. *vivax* (arrowhead); **D** Mature schizont of *P*. *vivax* (arrow); **E** Gametocyte of *P*. *vivax* (arrow); **F** Gametocyte of *P*. *falciparum* (arrow)

The annual parasite incidence (API) in Golestan Province for 2023 was recorded as 0.01 [10]. Low parasitemia was observed in 45.45% (10/22) of cases, intermediate parasitemia in 50% (11/22) of cases, and high parasitemia in 4.54% (1/22) of cases [9]. The mean ± standard error of mean (SEM) for the number of parasites/µL of blood sample was 16,029 ± 5,060 for *P*. *vivax* and 105,460 ± 102,146 for *P*. *falciparum* (Table 1). Among the patients, 77.27% (17/22) were infected with *P*. *vivax*, 18.18% (4/22) were infected with *P*. *falciparum*, and 4.54% (1/22) were co-infected with both *P*. *vivax* and *P*. *falciparum* (Table 1). For *P*. *vivax*, 5.55% (1/18) of the samples presented the ring form, 100% (18/18) trophozoite form, 33.33% (6/18) schizont form, and 16.66% (3/18) gametocyte form. In the case of *P*. *falciparum*, 20% (1/5) of the samples presented the ring form, 60% (3/5) trophozoite form, and 20% (1/5) gametocyte form (Fig. 3).

All patients were treated according to the national malaria treatment guidelines developed by the Iranian Ministry of Health. Chloroquine (CQ) along with primaquine (PQ) was administered to the patients infected with *P*. *vivax*. To eliminate the stages from trophozoite to blood schizont, chloroquine was prescribed at a dosage of 25 mg/kg over three days, consisting of 10 mg/kg on the first day and 7.5 mg/kg on the second and third days. Additionally, to eliminate the hypnozoites of *P*. *vivax*, primaquine was administered at a dosage of 3.5 mg/kg for 14 days, with a daily dose of 0.25 mg/kg [11]. Cases of *P*. *falciparum* and *P*. *falciparum*/*vivax* co-infection were treated as follows: artesunate for 3 days, 200 mg daily in two doses of 100 mg each along with 3 sulfadoxine-pyrimethamine tablets (500 mg sulfadoxine and 25 mg pyrimethamine) as a single dose with the first dose of artesunate. A single dose of primaquine (0.75 mg/kg, max 45 mg in adults) was administered to clear *P*. *falciparum* gametocytes and reduce transmission [12]. The patients were regularly monitored for clinical symptoms, and none of the patients exhibited signs of side effects related to the medications. The number of parasites in the patients’ blood samples was assessed on days 1, 2, 3, 7, 14, 21, and 28, and the adequate clinical and parasitological response (ACPR) was evaluated according to the World Health Organization protocol [11]. All patients demonstrated a positive response to the treatment, with a decrease in both the number of parasites and the number of clinical symptoms over time. Only the patient infected with *P*. *falciparum*, who exhibited a high level of parasitemia, was monitored over 42 days. Nevertheless, his blood smear showed negative results in less than 28 days.

## Discussion

Cases of malaria have been documented in Iran as far back as 1000 BCE [13]. Around the year 1000 AD, a deadly disease resembling malaria was prevalent in Gorgan city, Golestan Province, located in northeastern Iran, which is attributed to the humid climate. It was described as if the city of Gorgan had been overrun by the angel of death, earning it the ominous title of “the graveyard of the people of Khorasan Province”, who had migrated there from northeastern Iran [14]. Similarly, in Gilan Province, located in northern Iran, a proverb emerged that conveyed, “if you want to die, travel to Gilan” [15]. The high mortality rate attributed to malaria in the northern regions of Iran during this period suggests that the causative agent of these deaths was most likely the species *P*. *falciparum*. However, new evidence showed that *P*. *vivax* could cause severe disease and complications [16].

Approximately 1587–1629 CE, the Caspian Sea coastal region of Iran experienced a malaria epidemic of unparalleled magnitude, making it a pivotal event in the history of the country. This was the largest outbreak in Iran, resulting in the death of 20,000 individuals. Subsequently, malaria became endemic in the area, leading to high infection rates during the early 20th century [13]. The presence of numerous swampy areas and rice fields along the Caspian Sea coastal region, coupled with the existence of civil conflicts and the absence of a comprehensive and efficient healthcare system, played crucial roles in facilitating the spread of malaria during this period.

The earliest documented research on the prevalence of malaria in northern Iran was conducted in the cities of Rasht and Bandar-e Anzali, Gilan Province, in 1921 [13]. Subsequent studies were carried out in other areas of northern Iran. Research conducted between 1921 and 1942 along the Caspian Sea coast of Iran revealed the presence of *P*. *malariae*, *P*. *vivax*, and *P*. *falciparum* in the region, with most studies indicating *P*. *falciparum* as the predominant species, except in Golestan Province, where *P*. *vivax* and *P*. *malariae* were dominant [15]. Over time, the prevalence of malaria along the Caspian Sea coast decreased, with the last reported cases of *P*. *malariae* from this region dating back to 1998 [17]. In the present study, all identified cases were associated with *P*. *falciparum* and *P*. *vivax* species. A negative result by both microscopy and RDT is fairly reliable for excluding *P*. *falciparum* in most clinical settings. However, RDT, especially those detecting the HRP2 antigen of *P*. *falciparum*, can have false negatives due to factors like low parasite density, operator error, poor storage conditions, or genetic deletions of the PfHRP2/3 genes in the parasite [17–21].

In 1949, there was a significant increase in the number of reported malaria cases along the Caspian Sea coastal region of Iran, with daily reports ranging from 20 to 100 cases. Alarmingly, 30–40% of the deaths that occurred during that year were attributed to malaria, prompting the initiation of malaria control programs between 1949 and 1956. These efforts successfully removed the region from its hyperendemic status, leading to a notable decrease in malaria cases to only 10–15 cases per year by 1952 [22]. In 1956, a comprehensive program aimed at eliminating malaria in the Caspian Sea coastal region was subsequently implemented, ultimately resulting in the declaration of the Caspian Sea coastal provinces as malaria-free in 1961 [15].

In 1976, Golestan Province achieved malaria-free status, while the API in Mazandaran Province was less than 1, and in Gilan Province, it was less than 0.01 [23]. The occurrence of indigenous malaria cases in Golestan Province ceased before 1976, in Mazandaran Province before 1998, and in Gilan Province before 2007. Consequently, all reported malaria cases in these three provinces since then have been imported [18, 24, 25], which aligns with our findings.

**Fig. 4:**
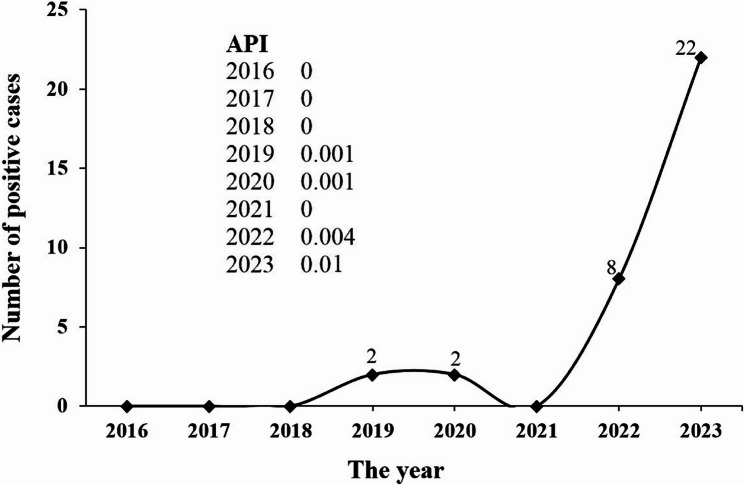
Malaria cases from 2016–2023 in Golestan Province, northern Iran. API: annual parasite incidence

In regions with high levels of malaria transmission, such as hyperendemic areas, the API tends to be elevated, increasing the risk of contracting the disease at a younger age [26]. In Golestan Province, the APIs over the past eight years have indicated a low risk of malaria transmission (Fig. 4), with the average age of infected individuals being 38 years in 2023, which aligns with the findings of other studies [27–29].

80% of reported human malaria cases in Iran are from the two provinces of Sistan and Baluchistan and Hormozgan Provinces [30–32], where 86% of the malaria cases reported in the current study originated from. The majority of infections (71.7–90%) are due to *P*. *vivax*, with *P*. *falciparum* comprising around 8–9% of cases [30–32], which is consistent with the results of our study. The aforementioned studies reported men are disproportionately affected (up to 87.1% of cases), and a large proportion of cases are among drivers and young to middle-aged adults [30–32]. This gender difference is attributed to greater exposure among men due to occupational and behavioral factors [31, 33]. In this research, the majority of the infected individuals were military personnel and drivers, who are traveling between provinces of the country. In Iran, almost all individuals working in the two identified professions are male, which explains why all the recorded positive cases were men. Furthermore, these two occupational groups spend a greater amount of time outdoors, consequently increasing their exposure to mosquito bites.

In the present study, the RDT and microscopic diagnosis methods were used to identify cases of malaria. The effectiveness of these methods can vary depending on the conditions. For example, the microscopic method has been shown to be more effective in diagnosing mixed infections compared to RDT [34]. The variation in the performance of these methods may be due to the severity of malaria in specific regions [35]. Therefore, the choice of diagnostic method may vary depending on the prevailing conditions in different areas. Most studies recommend polymerase chain reaction (PCR)-based methods for malaria diagnosis [36–40]. However, PCR is particularly recommended in hyperendemic areas where there may be asymptomatic carriers [41]. In Golestan Province, where there have been no endemic cases of malaria for a long time and all cases are imported with clinical symptoms, it is sufficient to use the microscopic diagnostic method along with RDTs, which is recommended by the World Health Organization (WHO) as the standard for malaria diagnosis. In this study, all positive cases with the microscopic and RDT methods were consistent, and there was no need for confirmation with other methods such as PCR. However, due to the regular malaria surveillance in Iran, in case of discordant results between the two mentioned methods, PCR is used for definitive confirmation.

*P*. *vivax* shows no confirmed chloroquine resistance in Iran [42]. Chloroquine remains the first-line treatment, with a mean parasite clearance time of 2.78 days [24]. *P*. *falciparum* developed resistance to chloroquine in southern Iran, with initial cases reported in 1983 (5.7% resistance rate) [42]. By 1996, resistance levels reached 51.2% in some regions [23], and recent studies show treatment failure rates as high as 78.5% [42]. This led to chloroquine being discontinued for *P*. *falciparum* treatment [23,42]. Currently, the first line of treatment for *P*. *falciparum* in Iran is artesunate along with sulfadoxine-pyrimethamine (SP) [43]. Nevertheless, resistance to SP emerged in southern Iran [42], However, SP remains partially effective when combined with other drugs like artesunate [43].

Over time, the criteria for evaluating the severity of malaria have changed. Factors such as parasitemia are now crucial in determining the disease condition and the appropriate treatment. A parasitemia level of over 100,000 parasites/µL is considered life-threatening [9]. In the present study, one *P*. *falciparum*-infected patient presented a parasitemia count of 411,852 parasites/µL and responded well to treatment, similar to other patients. This highlights the effectiveness of chloroquine along with primaquine against *P*. *vivax* and artesunate along with SP against *P*. *falciparum* in Golestan Province, northeastern Iran.

The presence of both male and female gametocytes in the peripheral blood of humans is crucial for the transmission of *P*. *falciparum* and *P*. *vivax* parasites to mosquitoes, with a ratio of 1:1 being necessary for the establishment of the life cycle. Increasing the ratio of female to male gametocytes by 4 to 1 enhances the likelihood of successful transmission [44, 45]. The aspects that play a role in the development and circulation of gametocytes consist of proteins released from maturing reticulocytes, the hematocrit level, the immunity of the host, the density of the parasites, and the exposure to antimalarial drugs [45, 46]. Among the cases presented, 4 out of 17 *P*. *vivax*-positive specimens showed the presence of gametocytes. In 3 of these samples, the ratio of female to male gametocytes was 1:1, whereas in 1 sample, it was 3:1. Furthermore, 1 out of 4 specimens of *P*. *falciparum* contained gametocytes, with a 1:1 ratio of female to male gametocytes. This finding suggests the potential for local transmission of the parasite in Golestan Province, northeastern Iran.

From 2018 to 2021, Iran did not report any cases of indigenous malaria. However, in 2022, 1,439 indigenous cases were reported, leading the WHO to declare it a malaria outbreak. However, there was doubt between introduced and indigenous cases in 2022 reported from Iran [47]. The surge in malaria cases in neighboring countries, Afghanistan and Pakistan, played a significant role in the spread of the disease to Iran. Pakistan experienced the greatest increase in malaria cases globally in 2022, with the number of cases increasing from approximately 500,000 cases in 2021 to approximately 2,655,000 cases in 2022, a fivefold increase [47]. Similarly, the number of malaria cases in Afghanistan also increased from approximately 82,748 in 2021 to approximately 288,000 in 2022 [47]. Climatic changes, including heat waves and catastrophic floods, fragile healthcare infrastructure, inadequate community education on malaria prevention, and population movement have contributed to the recent surge in malaria cases in both countries [48–50]. According to some reports, 47.42% of malaria cases in Iran were of Pakistani or Afghani nationality [51], and more than 90% of Afghan refugees in Iran were suffering from malaria [52], highlighting the impact of cross-border movements on disease spread. In the present study, 3 out of 22 people had traveled to Pakistan, and the other people had traveled to the southern and southeastern provinces of Iran, which borders the malaria-endemic countries of Pakistan and Afghanistan.

Seven specific species of Anopheles act as carriers of malaria, of which two species, *Anopheles maculipennis* and *Anopheles superpictus*, are found in Golestan Province. *Anopheles maculipennis* has been reported from two cities, and *Anopheles superpictus* has been reported from one of the 14 cities in this province [53]. This information is crucial, as it indicates a relatively low possibility of establishing local transmission of malaria in Golestan Province. Considering the increase in malaria cases in 2023 in this province, it is imperative to emphasize the importance of regular and accurate monitoring of malaria cases. Moreover, the vigilance of doctors and the health system in identifying infected individuals and recording positive cases is crucial. These measures will contribute to better regulation and implementation of malaria control and elimination programs in Golestan Province, as well as in Iran.

## Conclusions

Malaria has been a long-standing issue in Iran, with major outbreaks along the Caspian Sea coast in the past. Historical records suggest that these outbreaks were caused by the species *P*. *falciparum*. In the late 18th century, a significant epidemic in the region led to the death of 20,000 individuals and the subsequent endemicity of malaria. Control programs were implemented in the mid-20th century, successfully reducing malaria cases in the Caspian Sea coastal region. By 1961, the region was declared malaria-free. Golestan Province achieved malaria-free status in 1976, followed by Mazandaran and Gilan Provinces. However, imported cases of malaria have been reported since then, with an increase in malaria cases in 2022, potentially linked to cross-border movements from Afghanistan and Pakistan. This study discussed 22 cases of malaria diagnosed in 2023 in Golestan Province in northern Iran. Given the presence of malaria vectors in this area and the observation of gametocytes in specimens from some patients, the increase in reported malaria cases in this area could be worrisome in terms of establishing local transmission in this area.

## Data Availability

Data is provided within the manuscript.

## Abbreviations

HRP2: Histidine-rich protein 2
RDT: Rapid diagnostic test
ACPR: Adequate clinical and parasitological response
API: Annual parasite incidence
PCR: Polymerase chain reaction
WHO: World Health Organization
